# Level of client satisfaction among family planning service users in semi-pastoralist areas of Southeast Ethiopia: a mixed-methods study

**DOI:** 10.3389/fgwh.2024.1271115

**Published:** 2024-07-04

**Authors:** Hana Eshetu, Dawit Jember Tesfaye, Selam Fantahun, Bezawit Birhanu, Daniel Dere Deffecho, Shitalem Tadesse Teshager, Beka Teressa Meka, Zenawi Hagos Gufue

**Affiliations:** ^1^ School of Public Health, College of Medicine and Health Science, Hawassa University, Hawassa, Ethiopia; ^2^ Department of Health Policy and Management, Jimma University, Jimma, Ethiopia; ^3^ School of Medicine, College of Medicine and Health Science, Hawassa University, Hawassa, Ethiopia; ^4^ Department of Anaesthesia, College of Medicine and Health Science, Debre-Berhan University, Debre-Berhan, Ethiopia; ^5^ Department of Public Health, College of Health Sciences, Salale University, Fitche, Ethiopia; ^6^ Department of Public Health, College of Medicine and Health Sciences, Adigrat University, Adigrat, Ethiopia

**Keywords:** client satisfaction, family planning, mixed methods, semi-pastoralist, Ethiopia

## Abstract

**Background:**

Client satisfaction with family planning services is a crucial metric for gauging healthcare providers' performance. There is a dearth of local data that explores the factors that influence clients' satisfaction with family planning services in semi-pastoral areas using a mixed-methods approach. This study aimed to assess the level of client satisfaction and its associated factors among family planning service users in six public health centers in Southeast Ethiopia.

**Methods:**

A multi-centered, concurrent, mixed-method survey using quantitative and qualitative methods was conducted in six public health centers in Southeast Ethiopia from March 15 to April 16, 2022. Four hundred nineteen systematically selected family planning method users and their respective six family planning service providers were approached using a purposive sampling technique. A multivariable binary logistic regression model was used to identify the independent factors associated with clients' satisfaction with family planning services.

**Results:**

Four hundred fourteen study participants were finally approached, and client satisfaction with family planning services in those centers was 57.5% with a 95% CI of 52.71%–62.71%. Being in the age group of 25–34 years (AOR = 1.99; 95% CI 1.2, 3.29), married (AOR = 2.41; 95% CI 1.13, 5.15), waiting less than 30 min (AOR = 1.74; 95% CI 1.11, 2.72), and receiving the family planning method they want (AOR = 2.35; 95% CI 1.16, 4.76) were positively associated with client satisfaction. Updating the provider's skills and knowledge, keeping clients' method choices, and leaving free decisions also increased client satisfaction.

**Conclusions:**

In this study, client satisfaction with family planning services remains low. Users' age, marital status, waiting time, and wish to receive the method they want were positively associated with client satisfaction.

## Background

Client satisfaction with family planning services is a crucial indicator of how happy a client is with the service they receive from healthcare professionals. It also represents the discrepancy between the service that was received and what was anticipated by the client (1, 2). Donabedian asserts that client satisfaction is a fundamentally significant indicator of quality because it reveals if a provider was successful in exceeding the client's top priorities (3). Client satisfaction is a significant predictor of the quality of healthcare and is becoming a prominent outcome measure in most services (4).

Family planning counselors can discover the parts of care that matter most to their clients by focusing on the user's point of view, but they can also indicate opportunities for the service to be improved (5). Growing interest is being given to the assessment of clients' satisfaction with the delivery of family planning services in healthcare facilities as a tool for monitoring and evaluating primary healthcare programs in developing nations (6).

Studies have shown that sociodemographic factors, obstetric factors, and service delivery factors like waiting time, receiving sufficient information, privacy, the service's opening hour, receiving a description of the method's side effects, receiving a choice of method, and receiving instructions on how to use the method are all related to how satisfied clients are with family planning services (7–12). According to the studies conducted to assess the level of client satisfaction with family planning services, it was 33% in Pakistan (13) compared to 68% in Mozambique (14) whereas in Ethiopia it was 41.7% in Jigjiga (12) and 52% in Adama (8). These findings indicate that the level of client satisfaction with family planning services remains suboptimal.

To gauge customer satisfaction with family planning services in Ethiopia, several studies were carried out; however, there has been little research utilizing mixed methods, as many earlier studies done in Ethiopia focused on the quantitative study design to assess the level of client satisfaction and examine aspects that affect client satisfaction from the providers' perspective that have been documented, particularly in semi-pastoralist settings. To further understand the degree of customer satisfaction with family planning services, researchers are recommending the need for additional research utilizing mixed methods (11). Hence, this study aimed to assess the level of client satisfaction and its associated factors among family planning service users in six public health centers in Southeast Ethiopia.

## Methods and materials

### Study area and period

**Figure 1:**
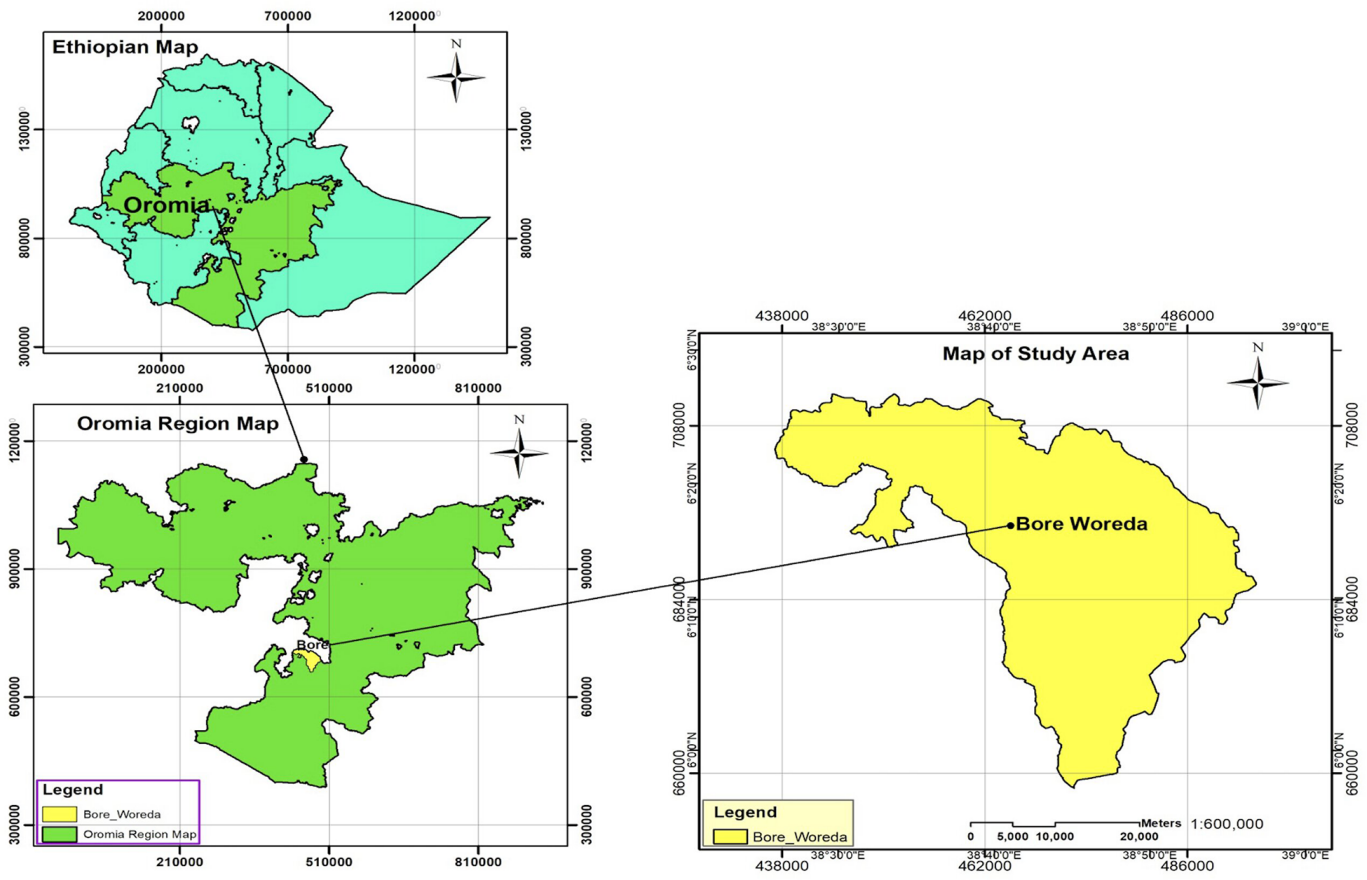
Map of the bore district, east Guji Zone, Oromia region, Southeast Ethiopia.

The study was conducted in the Bore district, East Guji zone, Oromia Region, Southeast Ethiopia. The Bore district is located 389 km southeast of Addis Ababa, the capital city of Ethiopia (Figure 1). The district had a total population of 210,179 according to the 2007 national census, of which 105,726 men and 104,453 women made up the population (15). In the district, there is one public primary hospital, six public health centers, and 33 health posts. The study was conducted in the six public health centers in the district from March 15 to April 16, 2022.

#### Study design

A multi-centered, concurrent, mixed cross-sectional study using quantitative and qualitative methods was conducted among family planning users and their service providers.

#### Study population

Systematically selected female family planning users who visited the district health centers and their service providers (who served at least 6 months) who were available during the data collection period were included in the study. Staff family planning users of the health centers and to avoid a double count, if the lady was previously interviewed and came for a family method shift or removal while we were collecting data, she was excluded from the study. Similarly, those who came for the removal of an implant or intrauterine contraceptive device and providers with less than 6 months' worth of experience were also excluded.

### Sample size determination

The sample size for the quantitative study was determined using the single population proportion formula by taking the proportion of clients satisfied with the family planning services, 46%, taken from the study conducted in Jimma (16), with a 95% confidence level and a 5% degree of precision. Accordingly, the calculated sample size was 381 and, after adding a 10% non-response rate, the final sample size was 419. Concerning the sample size for the qualitative study, one family planning service provider from each family planning unit of the six health centers (*n* = 6) was approached, and the sample size was determined by saturation of data.

### Sampling technique

**Figure 2:**
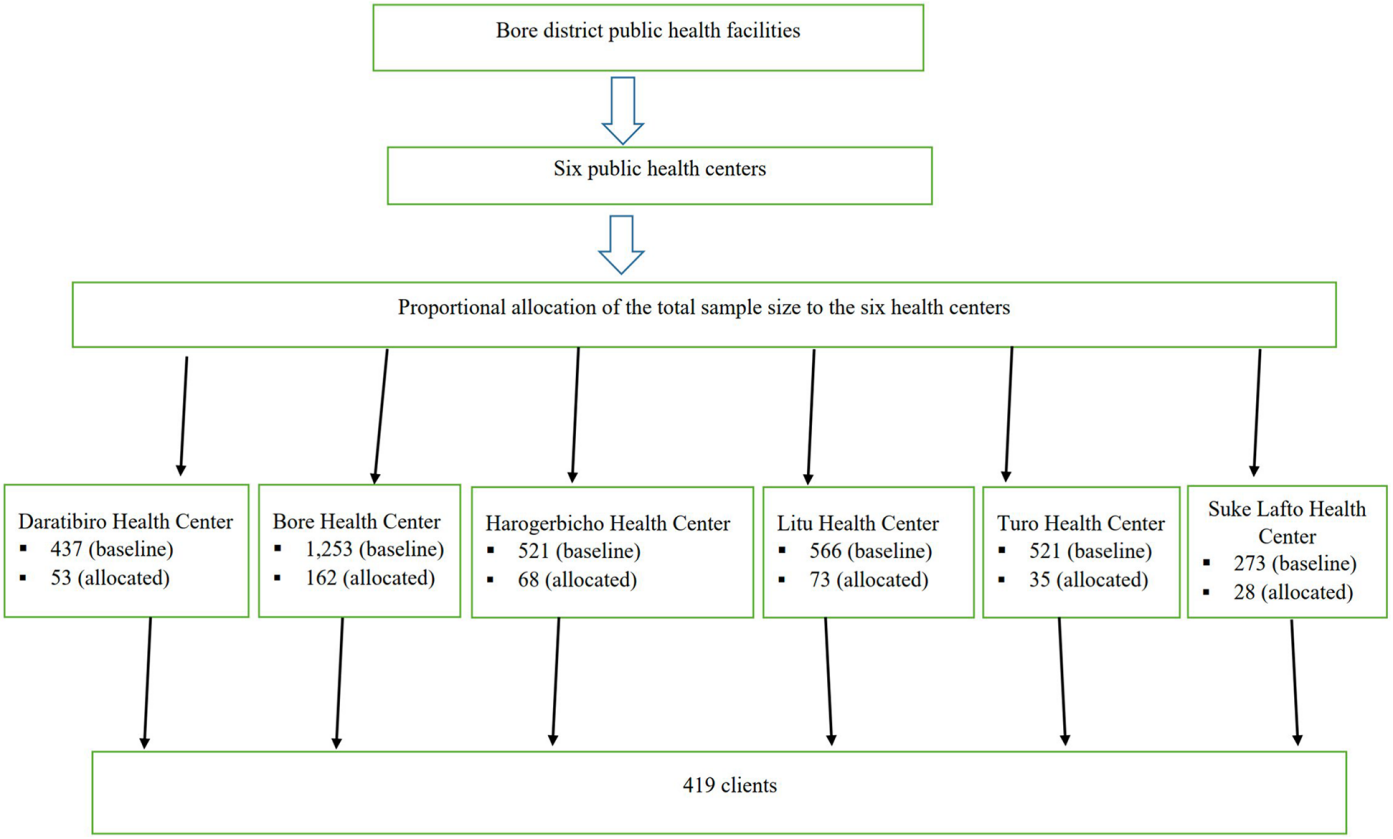
Schematic presentation of sampling technique among family planning service users in six public health centers in Bore district, East Guji Zone, Oromia region, Southeast Ethiopia.

For the quantitative study, a systematic random sampling technique was employed to recruit clients of family planning users from the six health centers. According to the 2021 and 2022 reports (17), a monthly average of 3,237 clients visited the family planning units of the six health centers. The calculated sample size was proportionally distributed based on the monthly flow of family planning users in each health center. The sampling interval was calculated by taking the assumptions (*k* = 3,237/419 = 8), and accordingly, every eighth client who visited the family planning unit of the respective health centers was included in the study (Figure 2). For the qualitative study, a purposive sampling technique was used based on the researcher's judgment regarding participants that were representative of the study phenomenon.

### Data collection tools and procedures

For the quantitative study, a pre-tested (among 5% of the sample size outside of the study area), structured, interviewer-administered questionnaire was used to collect the data. The data collection tool was adapted from a previous similar study (16) and contains the socio-demographic characteristics, service provision characteristics, obstetric factors, and status of service. The tool was originally prepared in English, then translated into the local language (Afan-Oromo) and re-translated back to English to maintain its consistency by an independent professional translator.

Six experienced nurses with bachelor's degrees and two experienced public health experts with master's degrees were recruited as data collectors and supervisors, respectively. The correctness of the responses, data collection instruments, and methods were all closely monitored daily. For the qualitative study, to collect pertinent information from the family planning service providers who have been working in the family planning service unit at those six health centers, a semi-structured interview guide was employed. The interviews were audio-recorded, and written notes were also used to record information.

#### Variables

The outcome variable was client satisfaction with family planning services (satisfied and unsatisfied), whereas, the independent variables were sociodemographic characteristics, service provision characteristics, obstetric factors, and status of service.

### Operational definitions

Client satisfaction: To gauge study participants' satisfaction with family planning services, 10 satisfaction-related items on a five-point Likert scale with values ranging from 1 (very unhappy) to 5 (extremely satisfied) were employed. The median score of satisfaction was used to dichotomize each respondent's satisfaction score. Accordingly, those who scored above the median were regarded as satisfied, while those who scored below the median level were judged unsatisfied with the family planning services (16). The amount of time a client had to wait before obtaining their services was referred to as the waiting time. Less than 30 min is considered the appropriate waiting time; any time over this mark is considered inappropriate (18).

### Data quality control

Data trustworthiness was appraised by the following criteria: credibility; participants were given enough time during the in-depth interview to express their opinions and experiences; they were interviewed in a welcoming environment; the principal investigator collected the data; and the data collector watched their facial expressions to decipher their nonverbal cues. Dependability, the same data collectors, and the same interview guide were utilized to conduct in-depth interviews to ensure the consistency of the data from all participants.

Following data collection, verbatim transcriptions of the raw or recorded data were made before they were translated into English. To ensure transferability and accurately represent the source population, in-depth interviews were conducted with members of the nominated sample. Conformability: to eliminate researcher bias during data collection, processing, and analysis, the participants' own words rather than the researchers' opinions and expectations were used. To minimize this, the data collectors were not dressed in white gowns, and they were hired from outside the study site.

### Data management and analysis

Every day, the surveys' consistency and completeness were reviewed. The gathered data were input into Epi Data version 4.6 software, and the entered data were exported to SPSS version 25 statistical software for analysis. Descriptive statistics, including frequency distribution, measures of central tendency, and variability, were computed. The dependent variable, which was the sum of the Likert scale questions, was dichotomized using the median as the cut-off point after the Shapiro-Wilk test was used to verify the normality of the data.

The bivariate analysis was done to check the existence of a crude association and to select candidate variables; those variables that are clinically important and have a *p*-value of less than 0.25 were included in the final model. Confounding was checked, and a percentage change in the regression coefficients of less than 20% reveals an absence of confounding. The interaction for the main effect model was also checked, and the partial likelihood ratio test result with a *p*-value > 0.05 and a variance inflation factor less than 10 indicated the non-existence of multi-collinearity among the independent variables.

The multivariable binary logistic regression model was used to identify the independent factors associated with clients’ satisfaction with family planning services. The summary measures of estimated crude odds ratios (COR) and adjusted odds ratios (AOR) with a 95% confidence interval were presented, and a *P*-value less than 0.05 was used to declare statistical significance. The goodness of fit of the model was assessed using the Hosmer and Lemeshow goodness of fit test.

We promptly translated and transcribed the verbatim audio recording and memo into English for preliminary analysis. To get a sense of what each study participant had said, the interview transcript was once again read line by line and listened to. The following questions that contained contradictory information were verified during an interview by the respondent's own words and double-checked with memoranda to ensure the accuracy of the data. Transcribed notes and audio were loaded into ATLAS ti version 8 for coding and classifying codes, and the output was exported into Excel and report form. Finally, the finding was clarified by combining categories and themes with the quantitative findings.

## Result

### Socio-demographic and obstetric characteristics

A total of 414 clients were included in the study, with a response rate of 98.81%, and 355 (85.75%) of the family planning service users were from rural areas. The median age of the respondents was 26 years, with an interquartile range of 22–30 years. One hundred twenty-four (30%) of the service users were unable to read and write and concerning marital status, 365 (88.16%) were married. Regarding the gravidity and parity of the service users, 233 (56.28%) and 258 (62.32%) were 1−3 gravidas and parous, respectively (Table 1)**.**

**Table 1:** Baseline characteristics of female family planning service users in the public health centers of Southeast Ethiopia, 2022 (*n* = 414).

Baseline characteristics	Frequency	Percent
Age, median (interquartile range), years	26 (22–30)	
Income, median (interquartile range), United States dollar	19.35 (9.67–48.36)	
Age group (in years)		
15–24	154	37.2
25–34	200	48.31
≥35	60	14.49
Residence		
Rural	355	85.75
Urban	59	14.25
Ethnicity		
Oromo	342	82.61
Sidama	36	8.7
Amhara	25	6.04
Others^a^	11	2.65
Religion		
Protestant	288	69.57
Orthodox	88	21.26
Waqefata	18	4.35
Muslim	14	3.38
Unspecified	6	1.44
Educational status		
Unable to read and write	124	29.95
Able to read and write	49	11.84
Primary school (grades 1–8)	117	28.26
Secondary school (grades 9–10)	59	14.25
Preparatory school (grades 11–12)	52	12.56
College and above	13	3.14
Marital status		
Married	365	88.16
Widowed	23	5.56
Divorced	20	4.83
Single	6	1.45
Occupational status		
Farmer	118	28.5
Housewife	77	18.6
Merchant	71	17.15
Private employee	50	12.08
Government employee	48	11.59
Student	34	8.21
Unemployed	16	3.87
Household monthly income (in United States dollars)		
<19.35	240	57.97
≥19.35	174	42.03
Gravidity		
Nulligravida	12	2.9
1–3	233	56.28
4–6	119	28.74
>6	50	12.08
Parity		
Nullipara	14	3.38
1–3	258	62.32
4–6	112	27.05
>6	30	7.25
History of unplanned pregnancy		
Yes	237	57.25
No	177	42.75

^a^ Others: Gurage, Silte, and Somale.

### Family planning service provision

Two hundred forty (58%) women used Implanon; only 3 (0.7%) were IUCD users, and 330 (79.71%) women were repeat users of family planning. The mean (± standard deviation) of the waiting times for clients before getting service was 30 ± 25 min. The mean (± standard deviation) of the travel time to the facility was 30 ± 40 min. Four hundred nine (98.8%) of the clients reported that there was enough privacy during consultation and examination, and 413 (99.8%) of family planning users believed that the provider kept their client information confidential.

Three hundred seventy-one (89.6%) were told how to use the method. Three hundred forty-two (82.6%) clients talked about other methods; 336 (81.2%) clients were counseled about the side effects of the method, and 310 (74.9%) were told what to do if they experienced any problems with the method before their next visit. Two hundred thirty-five (56.8%) of the clients said that the provider did not show them any printed materials on family planning during the discussion. The majority of the clients (98.6%) were told about their scheduled follow-ups and appointments (Table 2).

**Table 2:** Family planning services provided to the respondents in the public health centers of Southeast Ethiopia in 2022 (*n* = 414).

Service-related variables	Frequency	Percent
Family planning users		
Repeat	330	79.71
New	84	20.29
Waiting time (in minutes)		
≤30	182	43.96
>30	232	56.04
Travel time by foot to the health facility (in minutes)		
≤30	221	53.38
>30	193	46.62
Type of family planning method		
Implanon	240	57.97
Pills	89	21.5
Depo-provera	82	19.81
Intrauterine contraceptive device	3	0.72
Sex of the family planning service provider		
Male	122	29.47
Female	292	70.53
Enough privacy during consultation and examination	409	98.79
Told how to use the family planning method	371	89.61
Receiving the requested family planning method	357	86.23
Told about any other family planning method	342	82.61
Told about the method's side effect	336	81.16
Provider shows information, education, and communication materials	235	56.76
Received instructions on what to do in the event of an issue	310	74.88
Told your schedule and follow-up	408	98.55

### Client satisfaction

57.5% of clients were satisfied with the family planning services. The majority of the respondents were satisfied with the ease of getting to the clinic site (62.4%), waiting time (59.2%), opening hour convenience (62.1%), cleanliness of the clinic area (44.2%), the provider's friendly approach (71.1%), the information given to the client (70%), method availability (62.8%), maintaining privacy (78.8%), the provider's discussion on the client's health condition (73.6%), and the provider's knowledge (82.8%) (Table 3).

**Table 3:** Clients’ responses to satisfaction with family planning services in the public health centers of Southeast Ethiopia, 2022 (*n* = 414).

Satisfaction responses	Very dissatisfied	Dissatisfied	Neutral	Satisfied	Very satisfied
	Frequency (%)	Frequency (%)	Frequency (%)	Frequency (%)	Frequency (%)
The clinic site is easy to get to	1 (0.24)	19 (4.59)	136 (32.85)	170 (41.06)	88 (21.26)
Waiting time (waiting area)	5 (1.21)	7 (1.69)	157 (37.92)	175 (42.27)	70 (16.91)
Opening time convenience	1 (0.24)	9 (2.17)	147 (35.51)	182 (43.96)	75 18.12)
Cleanliness of the facility	4 (0.97)	57 (13.77)	170 (41.06)	112 (27.05)	71 (17.15)
Provider-friendly approach	2 (0.48)	8 (1.93)	110 (26.57)	206 (49.76)	88 (21.26)
Methods information	0 (0)	7 (1.69)	117 (28.26)	207 (50)	83 (20.05)
Sufficiency of the methods	1 (0.24)	26 (6.28)	127 (30.68)	169 (40.82)	91 (21.98)
Privacy during physical examination	1 (0.24)	5 (1.21)	82 (19.81)	185 (44.69)	141 (34.06)
Providers’ decision	1 (0.24)	17 (4.11)	91 (21.98)	196 (47.34)	109 (26.32)
Providers knowledge	3 (0.72)	34 (8.21)	34 (8.21)	203 (49.03)	140 (33.82)

### Factors associated with client satisfaction

The odds of those clients who were married were two times higher than those who were divorced or widowed (AOR = 2.41; 95% CI: 1.13, 5.15). The odds of those who receive the method they want being 2.3 times more satisfied than those who have not received the method they want (AOR = 2.35; 95% CI = 1.163, 4.756) Respondents with an age range of 25–34 were two times more satisfied than those whose ages were 15–24 years (AOR = 1.99; 95% CI: 1.20, 3.29) (Table 4)**.**

**Table 4:** Factors associated with the satisfaction of clients with family planning services in the public health centers of Southeast Ethiopia, 2022 (*n* = 414).

Variables	Satisfaction status		COR (95% CI)	AOR (95% CI)
	Satisfied	Unsatisfied		
	Frequency (%)	Frequency (%)		
Marital status				
Currently not-married	3 (1.3)	3 (1.7)	Reference	
Currently married	219 (92)	146 (83)	2.53 (1.32, 4.86)	2.41 (1.13, 5.15)*
Told how to use the method				
No	15 (6.3)	28 (15.9)	Reference	
Yes	223 (93.7)	148 (84.1)	2.81 (1.45, 5.44)	1.85 (0.85, 4.02)
Received the method you want				
No	17 (7.1)	40 (22.7)	Reference	
Yes	221 (92.9)	136 (77.3)	3.82 (2.08, 7.01)	2.35 (1.16, 4.76)*
Method side effect explained				
No	35 (14.7)	43 (24.4)	Reference	
Yes	203 (85.3)	133 (75.6)	1.87 (1.14, 3.08)	1.09 (0.58,2.07)
Information on what to do if problem arise				
No	46 (19.3)	58 (33)	Reference	
Yes	192 (80.7)	118 (67)	2.05 (1.31, 3.22)	1.44 (0.79, 2.63)
Sex of the service provider				
Female	157 (66)	136 (67.3)	Reference	
Male	81 (34)	40 (22.7)	0.57 (0.37, 0.89)	0.76 (0.46, 1.26)
Waiting time (minutes)				
>30 min	117 (49.2)	115 (65.3)	Reference	
<30 min	121 (50.8)	61 (34.7)	1.95 (1.30, 2.91)	1.74 (1.11, 2.72)*
Age group (years)				
15–24	77 (32.4)	77 (43.8)	Reference	
25–34	131 (55)	69 (39.2)	1.89 (1.23, 2.92)	1.99 (1.20, 3.29)**
≥35	30 (12.6)	30 (17)	1.00 (0.55, 1.82)	1.09 (0.55, 2.21)
Providers show information, education, and communication materials				
No	122 (51.3)	113 (64.2)	Reference	
Yes	116 (48.7)	63 (35.8)	1.70 (1.14, 2.54)	1.18 (0.71,1.96)
Occupational status				
Government employee	28 (11.8)	20 (1.4)	Reference	
Private employee	33 (13.9)	17 (9.7)	1.39 (0.61, 3.15)	1.62 (0.62, 4.24)
Merchant	33 (13.9)	38 (21.6)	0.62 (0.29, 1.29)	0.66 (0.29, 1.51)
Housewife	58 (24.4)	19 (10.8)	2.18 (1.01, 4.72)	2.27 (0.96, 5.34)
Unemployed	9 (3.8)	7 (4)	0.92 (0.29, 2.88)	0.99 (0.28, 3.59)
Student	16 (6.7)	18 (10.2)	0.63 (0.26, 1.54)	1.01 (0.35, 2.86)
Farmer	61 (25.6)	57 (32.4)	0.76 (0.39, 1.51)	0.79 (0.37, 1.71)

AOR, adjusted odds ratio; CI, confidence interval; COR, crude odds ratio.

* Significant predictors *p* < 0.05; **significant predictors *p* < 0.01.

## Qualitative study results

### Socio-demographic characteristics of the study participant

A total of six family planning service providers participated in a qualitative study. More than half of the study participants, 4 (66.6%) were males, and 4 (66.6%) of the study participants were aged between 30 and 35 years, with a mean age of 31. Four (66.6%) of the participants had less than 5 years of clinical work experience, and the mean of their work experience was 4.6 (Table 5). After coding and analysis of in-depth interview data, five themes emerged.

**Table 5:** Socio-demographic characteristics of family planning service providers in the public health centers of Southeast Ethiopia, 2022 (*n* = 6).

Baseline characteristics	Categories	Frequency	Percentage
Age (in years)	25–30	2	33.4
	>30	4	66.6
Sex	Male	4	66.6
	Female	2	33.4
Educational status	Diploma nurse	2	33.3
	Degree-holder nurse	3	50
	Master's holder professional	1	16.7
Work experience (in years)	<5	4	60
	≥5	2	40

### Theme I: training

The first theme that emerged from the data analysis was training, and the theme was categorized into three categories: those who take in-service training, the adequacy of training, and what kind of training is important to scale up client satisfaction among family planning service users.

### Receiving in-service training

The findings from the analysis of in-depth interview data revealed that almost all the participants take in-service training, except for one service provider. The providers respond whether the training was adequate or not Some responses are presented as follows:

*“I know a lot of things, and it is adequate.*” *[#p4, Female, 29 years old, MPH]*

### Important kinds of training

The participants were asked which kind of training was important: theoretical or practical Some respondents responded that it is better to give both theoretical and practical family planning training sessions to improve client satisfaction among family planning service users, while others responded that it is better to focus only on practical training, and one provider responded that comprehensive training is good. Their responses are presented as follows:

“*Both theoretical and practical training are important, but it is better to focus on the practical areas, specifically on long-acting family planning, where it is better to give practical training.*” *[#p2, male, 33 years old, BSC nurse].*

### Theme II: updated knowledge

The participants were asked whether they had updated knowledge to provide the family planning service. The second theme that emerged from the in-depth interview data analysis was up-to-date knowledge. Two categories were established under this theme: yes, I have updated knowledge, and no, I have not updated knowledge.

### Yes I have updated my knowledge

The majority of the respondents respond as they have updated knowledge and their responses are reported as follows:

“*Yes, I think I have up-to-date knowledge to provide the service.*” *(Participants #1, 3, 4, and 6).*

### I don't have updated knowledge

Some respondents responded that they don't think they have updated knowledge and their responses were reported as follows:

“*I don't know if there is any update since I took the training two years ago.*” *[#p2, male, 33 years old, BSC nurse] [#p5, female, 27 years old, diploma nurse].*

### Theme III: choice of method

The third theme that emerged was a choice of method, an appointment, shifting the method, and being told to take care of other health facilities. These findings implied that client satisfaction among family planning service users can be scaled by considering the client's choice of method, a feasible appointment, and having a one-stop center that can deliver all the family planning methods without referring to other health facilities. The participants were asked about their response if there is no choice of method for a client. According to the findings, among the participants who took part in this study, some of them responded as follows:

### Appointment

“*I will describe the available methods, and if there is no method that she wants, the option that I have is to advise her to take other available methods. Other than this, I will appoint her by advising her to use the natural method.”* [#p2, male, 33 years old, BSC nurse].

### Shifting the method

Three of the family planning service providers responded that they would shift the method, and their responses are presented as follows:

“*If a client comes to my clinic and there is no method that she wants, first I will describe the method that is available right at that time. I elaborate on both the effectiveness and the side effects; therefore, I will inform her that this is all in my hands, and then, if she refuses to accept, I will not force her to take it, but if she accepts, I will give her the available method.”* [#p1, male, 32 years old, diploma nurse].

### Recommending another health facility

“*For example, a client may need to take Depo-Provera but there is no Depo-Provera so I will assure her to use other types of Methods by describing both the benefit and the side effects to assure her to use the available method but if she refuses, I will link or recommend her to go to other facilities; it may be a hospital where the method will be available*” *[#p4, female, 29 years old, MPH].*

### Theme IV: factors influencing client satisfaction

The participants were asked about factors that influence client satisfaction with family planning services. Different factors influence client satisfaction with family planning services from different health provider's perspectives. These are lack of knowledge and understanding about family planning, religious factors, cultural factors that promote having more children, lack of trained health providers, bad attitude of the community, male involvement, lack of short-acting family planning method, and availability and accessibility issues are some factors that affect client satisfaction with family planning service. This finding was revealed by the following sampled responses:

“*In my opinion, the majority lack knowledge and understanding about family planning, religious factors, and culture because the culture and religion do not support family planning methods, which may decrease the quality. And, particularly in rural areas, the cultural system does not support family planning because people want to have more children*” *[#p1, male, 32 years old, diploma nurse].*

## Discussion

This study assessed the level of client satisfaction with family planning services and associated factors in a semi-pastoralist area of Southeast Ethiopia using a mixed method. In this study, client satisfaction with family planning services is 57.5%. In this study, two groups of factors affecting clients' satisfaction with family planning services were identified. The first group of factors was related to marital status and age. The second factor was service-related, such as waiting time to receive the family planning service and obtaining a method of choice. These findings provide useful suggestions for improving the quality of family planning services through interventions that improve client satisfaction.

This level of client satisfaction is high when compared to the study done in Pakistan at the Jinnah Hospital in Lahore (33%) (19). This discrepancy might be due to differences in sample size and study setting. Similarly, a study conducted in Tembaro district and Jigjiga city found 41.7% and 46%, respectively (7, 12). This discrepancy might be due to socio-demographic differences in judging client satisfaction because the study done in Jigjiga only used six Likert scale item questions and the study done in Tembaro used fifteen Likert scale item questions, whereas in this study ten Likert scale item questions were used to assess the level of client satisfaction.

Another study that was conducted in Jimma Hospital shows the level of client satisfaction was 46% (16). This might be due to the difference in sample size and client expectations because the study done in Jimma was done in a hospital setting. However, this result is lower when compared with the study done in Kucha 68.4% (11), Kersa 68.8% (20), southern Ethiopia 67% (21), Hosanna (76.3%) (22), Bahirdar 66.1% (9), Tanzania 91% (6), Sokoto, northern Nigeria (85%) (10), Mozambique 86% (23), and Mexico 80% (5).

This discrepancy may explain the difference in the study period as a result of customers' higher expectations for the services they will receive due to the rapid advancement of technology and people's thinking. Additionally, this discrepancy may be the real difference in the availability of different contraceptives, as evidenced during this study when the stock of Depo-Provera was low and absent in some public health centers and some clients did not receive the method they wanted. In this study, the choice of method was significantly associated with client satisfaction, which may be due to different socio-demographic characteristics in those studies done in Ethiopia.

In this study, attempts were made to find out the most important contributing factors for client satisfaction with family planning services in the literature. Though the cause-and-effect relationship could not be established, comparable results were reported in other studies. The level of client satisfaction with family planning services was significantly associated with marital status. In this study, the odds of those who were married being satisfied were two times higher than those who were divorced or widowed. This is similar to the study done in Lagos, Nigeria (24).

This may be because sexual intercourse outside of marriage is a norm, culturally unacceptable, and religiously sinful, so there may be provider discrimination and a bad attitude toward clients who are not married but seek contraceptives. Also, the finding was best supported by qualitative findings, which stated that those married participants were urged by their husbands not to use long-acting family planning. This implies that male involvement in those married participants is more satisfied by method choice through intensive discussion.

In this study, the odds of those aged 25–35 being two times more likely to be satisfied than those whose ages were 15–24 were two times higher. This is consistent with the study conducted in Jimma health centers (18) and with the study conducted in Kucha (11). This may be because most clients use the service in this age group. Our study was contradictory to the studies done in Kenya (25), Egypt (26), and Mexico (5). This may be due to the different socio-demographic characteristics of the study area.

The odds were that those who got the method they wanted were more likely to be satisfied than those who did not get the method they wanted. This is similar to the study conducted in Jimma Zone health posts (27), Sodo Gurage Zone (21), Sokoto, Northern Nigeria (10), Lagos, Nigeria (24), and Mexico (28). This may be because, most of the time, people are satisfied when they get their choice. The odds of those who got service within 30 min being satisfied were higher than those who were waiting more than 30 min. This is similar to the study conducted in Jimma Hospital (16), Jimma Health Centers (18), Southern Ethiopia (21), Hosanna (22), Adama (8), Tembaro district (7), Bahirdar (9) and Mexico (28). This may make people satisfied when the service is provided without delay.

### Strengths and limitations of the study

A mixed approach was used, which gives a broader perspective than using a single-study approach. The study gives a clear understanding of the problem from different dimensions. So, the study was internally valid and consistent since the findings were triangulated to validate the quantitative study with a qualitative study. There may be courtesy bias, and as a result, clients may respond positively to positive answers. There may also be a Hawthorne effect, and providers may change their usual working patterns and work accordingly when they know the client is being interviewed about the service.

## Conclusions

The overall level of client satisfaction with family planning services was low in this study area compared to other studies. Marital status, age, waiting time, and receiving methods of choice were predictor variables that had statistically significant associations with client satisfaction with family planning services. Also, updating family planning providers both in practical and theoretical sessions, male involvement, and keeping the clients' choice of method, unlike service provider interference, were understood as characteristics that increase client satisfaction in family planning utilization. We recommended relevant stakeholders strengthen the supply of both long-acting and short-acting family planning methods regularly. Capacity building should be strengthened, family planning service training should be given comprehensively, and facilitating the service efficiently should shorten the client waiting time.

## Data availability statement

The original contributions presented in the study are included in the article/Supplementary Material, further inquiries can be directed to the corresponding author.

## Ethics statement

Ethical approval was obtained from the Institutional Review Board of the College of Medicine and Health Sciences, Hawassa University (IRB/071/14). Verbal informed consent was obtained from each participant, the collected data were handled with strong confidentiality, and all the collected information was stored anonymously. The study was conducted following the 1964 Declaration of Helsinki.
